# Optomotor-Blind Negatively Regulates *Drosophila* Eye Development by Blocking Jak/STAT Signaling

**DOI:** 10.1371/journal.pone.0120236

**Published:** 2015-03-17

**Authors:** Yu-Chen Tsai, Stefan Grimm, Ju-Lan Chao, Shih-Chin Wang, Kerstin Hofmeyer, Jie Shen, Fred Eichinger, Theoni Michalopoulou, Chi-Kuang Yao, Chih-Hsuan Chang, Shih-Han Lin, Y. Henry Sun, Gert O. Pflugfelder

**Affiliations:** 1 Institute of Genetics, National Yang-Ming University, Taipei; Institute of Molecular Biology, Academia Sinica, Taipei, Taiwan, Republic of China; 2 Department of Life Science and Life Science Center, Tunghai University, Taichung, Taiwan, Republic of China; 3 Theodor-Boveri-Institut, Biozentrum, Lehrstuhl für Genetik und Neurobiologie, Universität Würzburg, Am Hubland, Würzburg, Germany; 4 Institut für Genetik, Universität Mainz, Mainz, Germany; 5 Department of Entomology, China Agricultural University, Beijing, China; LMCB, University College London, UNITED KINGDOM

## Abstract

Organ formation requires a delicate balance of positive and negative regulators. In *Drosophila* eye development, w*ingless* (*wg*) is expressed at the lateral margins of the eye disc and serves to block retinal development. The T-box gene *optomotor-blind* (*omb*) is expressed in a similar pattern and is regulated by Wg. Omb mediates part of Wg activity in blocking eye development. Omb exerts its function primarily by blocking cell proliferation. These effects occur predominantly in the ventral margin. Our results suggest that the primary effect of Omb is the blocking of Jak/STAT signaling by repressing transcription of *upd* which encodes the Jak receptor ligand Unpaired.

## Introduction

The *Drosophila* compound eye originates from the eye-antenna anlage in the embryo. These cells proliferate and form the eye-antennal disc in the larva. In the mid-third instar eye disc, a wave of cell cycle coordination and apical cellular constriction, called the morphogenetic furrow (MF) forms at the posterior margin and progressively moves toward anterior. Posterior to the MF, retinal cell fates are specified by a series of cellular interactions [1,2,3,4]. The early steps of eye development involve at least three aspects: specification of eye fate, control of cell proliferation, and initiation and progression of the MF.

A large number of genes are involved in promoting eye development. Eye fate is specified by the retinal determination gene network which includes the transcription factors encoded by *eyeless* (*ey*), *twin of eyeless* (*toy*), *sine oculis* (*so*), *eyes absent* (*eya*), and *dachshund* (*dac*) [5,6]. Cell proliferation is highly regulated. Undifferentiated cells anterior to the MF undergo proliferation that is promoted by Notch signaling, the Pax protein Eyg, a combination of the transcription factors Eyeless, Homothorax (Hth), Teashirt (Tsh) and the transcriptional coactivator Yorkie (Yki), as well as Upd/Jak/STAT signaling [7,8,9,10,11,12,13,14]. MF initiation and progression are promoted by the Decapentaplegic (Dpp), Hedgehog (Hh) and Upd/Jak/STAT signaling pathways [11,15,16,17,18,19,20,21,22,23,24].

However, developmental processes rarely proceed by agonistic action alone but tend to be held in check by interaction between agonists and antagonists. The necessity to keep retinal development in bounds is obvious in the eye-antennal imaginal disc since this disc, in addition to the retina, gives rise to much of the exterior of the adult head [25,26,27]. Molecules with the ability to block eye development include Patched (Ptc) and other negative regulators of Hh signaling, Wingless (Wg) and the positive components of its signaling pathway, the transcription factors and cofactors encoded by *homothorax* (*hth*), *teashirt* (*tsh*), *hairy* (*h*), *extramacrochaetae* (*emc*), *pannier* (*pnr*), *Chip*, a*rrowhead* (*awh*) and *Lim1* [6,13,28,29,30,31,32,33,34].

Of these anti-retinal genes, Wg is the only signaling ligand and appears to be the most important anti-retinal factor. In the third instar eye disc, *wg* is expressed in the lateral margins and prevents inappropriate marginal morphogenetic furrow initiation [30,35]. Wg exerts its anti-retinal function by several routes. First, Wg blocks MF initiation [30,35]. A primary target is Dpp, which is essential for MF initiation [15,36]. Wg signaling represses *dpp* transcription and Dpp signaling at a step downstream of receptor activation [37,38]. Second, Wg also blocks MF progression [35] and neuronal differentiation through repression of Daughterless (Da) [38].

Which gene is induced by Wg to mediate its anti-retinal functions? One prime candidate is *optomotor-blind* (*omb*, FlyBase *bifid*, *bi*) which is expressed in the lateral margins in a pattern similar to the *wg* expression domain [39]. Ectopic expression of either *wg* or its downstream effector *armadillo* (*arm*) induces the expression of *omb* near the lateral margins [40]. *Omb* encodes a T-domain transcription factor and is required for the development of the optic lobes, wing, abdomen, and terminalia [41,42,43,44,45,46,47,48,49,50,51]. The polar eye disc expression and the fact that ectopic *omb* can completely block eye development [52] led us to investigate the role of *omb* in this process, and its relationship with Wg.

We show that Omb antagonizes eye development primarily at the level of cell proliferation. We further identified a molecular pathway downregulated by Omb. Our results suggest that the main effects of Omb are a block of Jak/STAT signaling by suppressing transcription of *upd* encoding the Jak/STAT ligand Unpaired. The block of Jak/STAT signaling accounts for the effect of Omb on cell proliferation. Our results also show that Omb mediates part of the Wg anti-retinal effects.

## Materials and Methods

### Fly stocks

Fly culture and crosses were performed according to standard procedure at 25°C unless noted otherwise. Transgenic expression lines: *UAS-omb* and *hsp70-omb* [47], *UAS-arm* [40], *UAS-dpp* [53], *UAS-upd*, *UAS-hop* [54]. *STAT92E* ^*397*^[55], *STAT92E* ^*06346*^ (*STAT* ^*P1681*^-*lacZ*, in [56,57]). *dpp* ^*C40*.*6*^-*GAL4* [53], *omb* ^*P3*^-*GAL4* (*GAL4-bi* ^*md*^ *653* in [58]; cf. [59], *ey-GAL4* [37] and *omb* ^*P7*^-*GAL4* (*omb3* in [60]; cf. [59] were used as *GAL4* drivers. Alleles used are: *omb* ^*bi*^ (regulatory hypomorph, [41]), *l(1)omb* ^*D4*^, *l(1)omb* ^*3198*^, and *l(1)omb* ^*15*^ (molecularly defined null mutants, [61,62]), *omb* ^*For*^ (gain-of-function mutant caused by a large downstream insertion [51]) and *omb* ^*P7*^-*GAL4* is hemi- and homozygous lethal and was used both as an *omb* allele and as *GAL4*-driver in the *omb* expression domain [60]. *lacZ* reporter lines are: *omb-lacZ* (*omb* ^*P*^ *1* in [63]), *dpp-lacZ* (BS3.0)[3], *mirr-lacZ* [63], *fng-lacZ* [64], and *wg-lacZ* [65]. *w omb* ^*P*^ *1 l(1)omb* ^*3198*^ was obtained by intragenic recombination. *dpp-lacZ (BS3*.*0)*, *dpp-GAL4c* ^*40*.*6*^ and *wg* ^*IL114*^ were kindly provided by Jessica Treisman, *omb* ^*For*^ by Marc Fortini, *Act5C>CD2>GAL4* [23], *tub>CD2>omb* and *tub>CD2>GAL4* by Christian Dahmann. Other fly stocks were obtained from the Bloomington *Drosophila* Stock Center and the Mid-America *Drosophila* Stock Center (Bowling Green, Ohio).

### Construction of 10X STAT-GFP-nls

GFP-nls sequence was amplified from pH-Stinger [66] by PCR primers (GGTTCAGGGGGAGGTGTGGG; ACTCGAGGCAGCCAAGCTGATCCTCTAGGG) and then cloned into 10XSTAT-luciferase [66] by *Xho* I and *Xba* I to generate the10X-STAT-GFP-nls construct. Germline transformants were generated as described previously [67].

### Clonal induction

Positively labeled flp-out expression clones were generated by crossing *UAS*-lines to *hs-FLP* ^*122*^; *Act5C>y* ^*+*^ *>GAL4 UAS-GFP* ^*S65T*^ [68]. Heat shock induction of *hs*-*FLP* ^*122*^ was at 37°C for 30 min at 24–48 hr after egg laying. *l(1)omb* ^*D4*^ and control clones were generated by incubating *hs-flp* ^*122*^ *hs-GFP FRT19/FRT19* or *hs-flp* ^*122*^ *hs-GFP FRT19/l(1)omb* ^*D4*^ *FRT19* larvae at 48–60 h AEL at 38°C for 30 min. Larvae were raised at 25°C for 48 h. Before dissection, larvae were subjected to 37°C for 1h and then shifted back to 25°C for 1h to allow GFP expression. *omb* gain-of-function and control clones were generated by incubating *hs-flp* ^*122*^; *tub>CD2>GAL4; UAS-GFP* or *hs-flp* ^*122*^; *tub>CD2>omb; UAS-GFP* larvae at 36–48h AEL for 30 min at 37°C. Larvae were dissected after 72 h at 25°C.

### Immunohistochemistry

Late third instar larval imaginal discs were dissected and stained. Primary antibodies were rat anti-Elav 7E8A10 (1:500, Developmental Studies Hybridoma Bank, U. of Iowa (DSHB, Iowa), rabbit anti-ß-galactosidase (1:1000, Cappel), mouse anti-Eya 10H6 (1:200, DSHB, Iowa), rabbit anti-BarH1(S12) (1:1000, gift from Tetsuya Kojima), rabbit anti-Omb (1:1000, [47,49]), rabbit anti-phospho-histone H3 (anti-PH3) (1:200–1:1000, Upstate Biotechnology), rabbit anti-Caspase-3 (cleaved) (1:200, Upstate Biotechnology), mouse anti-CD2 (rat) (1: 2000, Serotec), and mouse anti-Wg 4D4 (1:200, DSHB, Iowa), mouse anti-BrdU (1:50, Roche). Secondary antibodies (Jackson ImmunoResearch) were FITC-, Cy3- or Cy5-conjugated anti-rabbit, anti-rat and anti-mouse. Confocal microscopy was performed on a Zeiss LSM 310 or LSM 510. X-Gal staining of *lacZ* expression was done as described [63]. Anti-BrdU staining was performed as described [9].

### RNA *in situ* hybridization

*upd* RNA *in situ* hybridization is executed as described [9].

### Tissue sections

Semi-thin plastic eye sections were performed according to [69].

### Scanning electron microscopy and determination of ommatidial number

Scanning electron micrographs of adult eyes were obtained as described [52]. Due to the curvature of the eye, the ommatidial number N cannot be obtained from a single micrograph. A given eye was photographed from different angles. Dust particles or aberrations in the bristle pattern allowed the alignment of the otherwise repetitive structures such that error-free counting was possible.

## Results

### *omb* negatively regulates eye size

In the eye imaginal disc, Omb is expressed in two cell types. Within the main epithelium, Omb is expressed at the dorsal and ventral margins (S1A Fig., arrow). Omb is also expressed in the retinal basal glial cells that lie at the basal level of the eye disc and in the optic stalk [39,70] (S1A Fig.). Only the epithelial expression will be considered here. We found that loss-of-function and gain-of-function *omb* mutations caused changes in eye size.

In *omb* hypomorphic allele combinations and *omb* knock-down, the Omb level was reduced in both margins and the eye disc was enlarged (S1B Fig.). This was observed for the *omb* hypomorph *omb* ^*bi*^ in combination with any of three molecularly characterized *omb* null alleles, *l(1)omb* ^*D4*^, *l(1)omb* ^*282*^, and *l(1)omb* ^*3198*^ [61,62]. In these adults, the eye was enlarged with an increase in ommatidial number (N) by up to 25%, from 750–800 in wild type to 850–1000 in the mutant (wild type: N = 782, SD = 11.5, n = 6; *omb* ^*bi*^ */l(1)omb* ^*282*^: N = 952, SD = 61, n = 9).

**Fig 1:**
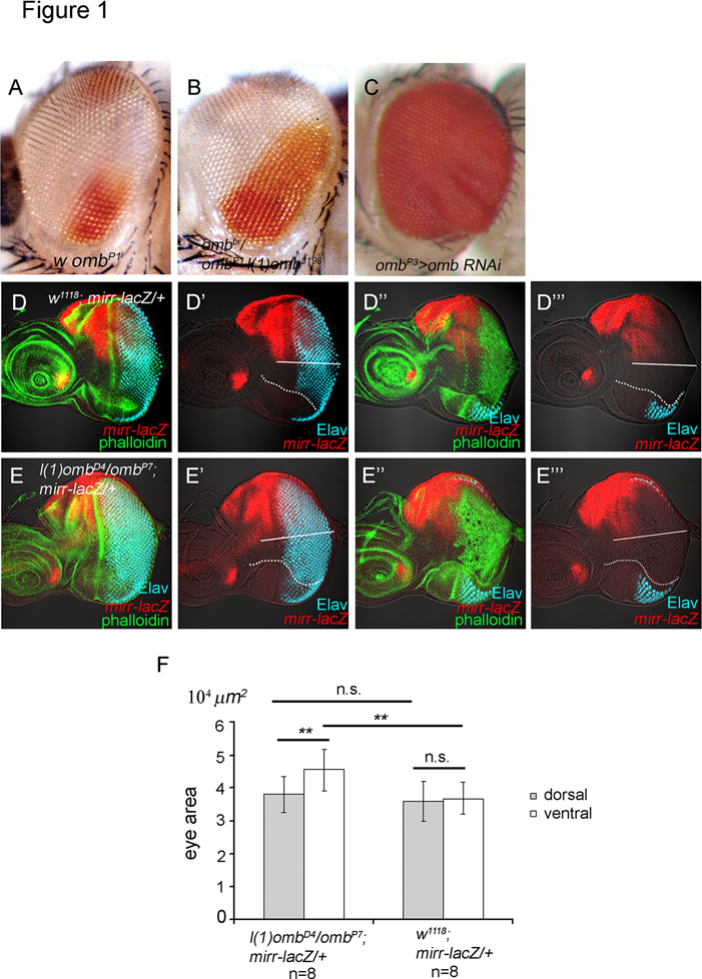
*omb* expression level influences eye size. (A) *w omb* ^*P1*^ (an enhancer trap insertion that does not affect *omb* expression and function, Sun et al., 1995), (B) *w omb* ^*P*^ *1 l(1)omb* ^*3198*^ */w omb* ^*bi*^. The expanded territory of ventral eye fate is clearly evident. Because of the increased size, the eye surface is more convex. Therefore, the unaffected dorsal pigmentation is not fully visible under this angle. (C) *omb* ^*P3*^ *>omb-RNAi* showed strong overgrowth in the eye. The overgrowth is stronger in the ventral than in the dorsal part of the eye. The eye is convoluted. (D-E) *mirr-lacZ* (anti-beta-galactosidase, red). Phalloidin staining (green). Elav (blue). (D-D”’) *mirr-lacZ/+* eye disc showing the dorsal-specific expression of *mirr-lacZ*. D, D’ and D”, D”’ are two focal planes. The D”, D”’ focal plane shows the ventral flap. (E-E”’) *l(1) omb* ^*D4*^ */ omb* ^*P7*^ eye disc. E, E’ and E”, E”’ are two focal planes. The E”, E”’ focal plane shows the ventral flap. The dorsal and ventral eye regions were distinguished (separated by a white line) based on *mirr-lacZ* and the position of the optic stalk. Two different focal planes are acquired in each eye disc. The area of eye disc including the ventral flap, based on two focal planes, were measured by the software, Zeiss Zen 2009. The results are summarized in (F). The ventral area of *l(1)omb* ^*D4*^ */omb* ^*P7*^; *mirr-lacZ/+* are significantly enlarged compared to that of *mirr-lacZ/+*. The dorsal area of *l(1)omb* ^*D4*^ */omb* ^*P7*^; *mirr-lacZ/+* are not significant increased compared to that of *mirr-lacZ/+*. Differences (*) presented in (E) and (J) are significant (Student′s t-test, **, p<0.05; *n*.*s*., non-significant). In all panels anterior is left and dorsal up.

The expansion of the eye occurred primarily ventrally. The dorsal-ventral distinction was based on several criteria. Using the enhancer trap insertion *omb* ^*P1*^ [63] as marker for dorsal and ventral ommatidia (Fig. 1A), only an increase in the ventral expression domain could be observed in the adult eye of *omb* hypomorphs (Fig. 1B). In the larval eye disc, the location of the dorsoventral (DV) midline was defined by the location of the optic stalk (S2A-D, Fig., arrowhead), the inversion of ommatidial chirality based on anti-Bar staining [71] (S2A-B Fig.), the ventral-specific *fng-lacZ* expression [64] (S2C-D Fig.) and the dorsal-specific *mirr-lacZ* expression (Fig. 1D, E). There was an obvious enlargement of the ventral eye disc in *l(1)omb* ^*D4*^/*omb* ^*P7*^ hypomorphic larvae (Fig. 1D-F; S2B, D Fig.). We followed the developmental progress of *omb* ^*P7*^ eye discs, based on the number of ommatidial rows, and found that the number of ommatidia in the ventral region was consistently higher relative to that in the dorsal region, which was not different from wild type (S2E Fig.). By all these criteria, an overgrowth of the ventral relative to the dorsal part was evident in the *omb* hypomorphic mutant eye.

These loss-of-function effects were also observed when *omb* was knocked down by RNAi. Expressing *omb*-*RNAi* [48] in its own expression domain using *omb* ^*P3*^-*GAL4* caused a strong reduction of Omb level in the margins of eye disc, the retinal basal glia and in the antenna disc This resulted in a strong overgrowth of eye disc (S1B Fig.) and adult eye (Fig. 1B). When *omb-RNAi* expression was driven by GMR-*GAL4*, the size of eye disc and adult eye was normal (not shown). This is consistent with *omb* expression not overlapping with the activity of the GMR-*GAL4* driver, which is restricted to cells posterior to the MF [72].

In contrast to the loss-of-function effects, gain-of-function of *omb* caused reduction or elimination of the eye. In the regulatory dominant gain-of-function allele *omb* ^*For*^ [51], Omb was overexpressed in the lateral margins and in the retinal basal glia (S1C Fig.). *omb* ^*For*^ larvae had smaller eye discs (S1C Fig.) and the adults had a reduced number of ommatidia and a posterior indentation in the eye (wild type: N = 782, SD = 11.5, n = 9; *omb* ^*For*^: N = 507, SD = 57.6, n = 6). Targeted mis-expression of *omb* in the lateral and posterior margins by *dpp-GAL4* (*dpp>omb*) causes a strong reduction or total absence of the adult eye [52]. Specific overexpression of *omb* at the lateral margins using *30A-GAL4* [73] caused a decrease in ommatidial number N that depended on the strength of the *UAS-omb* line (*UAS-omb* ^*4–15*^: N = 601.9, SD = 52.3, n = 10; *UAS-omb* ^*2–17*^: N = 670, SD = 32.3, n = 9).

In summary, the loss and gain-of-function phenotypes of *omb* indicate that *omb* is a negative regulator of eye development. The effect is stronger on the ventral side of the eye.

### *omb* blocks cell proliferation during eye development

Deviations from normal eye size can arise by several mechanisms. Changes in proliferation, cell death, morphogenetic furrow progression or retinal differentiation can all affect eye size. We tested the effect of loss and gain of *omb* on proliferation and cell death.

**Fig 2:**
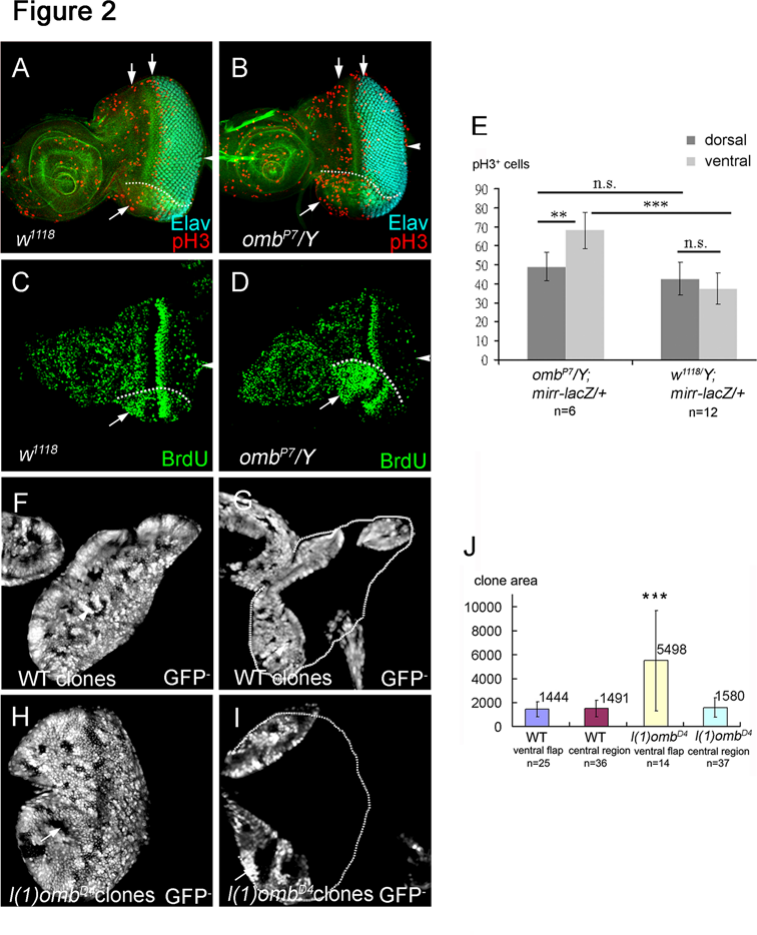
Omb blocks cell proliferation in eye disc. Cell proliferation was monitored by staining against the mitotic marker phospho-histone 3 (pH3), BrdU incorporation, and by comparing clone size in late third instar eye discs. (A-D) Arrowhead points to the position of the optic stalk. (A) Wild type eye disc showing the two mitotic waves (arrows) labeled by anti-pH3 (red). (B) The *omb* ^*P7*^ mutant showed an increased number of pH3-positive nuclei in the ventral eye compared to wild type. (C, D) BrdU incorporation showed an increase of proliferating cells in the ventral flap (arrow) of the *omb* ^*P7*^ mutant eye disc (D) compared to the wild type eye disc (C). (E) *mirr-lacZ* was used to mark the dorsal region. pH3 positive cells were scored in *omb* ^*P7*^ */Y; mirr-lacZ/+* and *mirr-lacZ* dorsal and ventral eyes. In order to include the ventral flap area, the images of several optical sections were merged. The quantification results are summarized in (E). The mitotic cells in ventral eye of *omb* ^*P7*^ is significant increased compared to ventral eye in wild type (p<0.05). (F, G) A wild type eye disc with clones (marked by the absence of GFP) at two focal planes to show the central region (F) and the ventral and dorsal flap regions (G). The clones were of similar size in all regions (summarized in J). (H, I) An eye disc with *l(1)omb* ^*D4*^ clones (marked by the absence of GFP) at two focal planes to show the central (H) and ventral and dorsal flap regions (I). The wild type clones and *l(1)omb* ^*D4*^ mutant clones were induced at the same time. The *l(1)omb* ^*D4*^ clones in the ventral flap were on average about 3.5 times larger than *omb* clones in the central region of the disc or than wild type clones (summarized in J). Differences (*) presented in (E) and (J) are significant (Student′s t-test, *** p<0.001; **, p<0.05).

In *omb* hypomorphs, cell cycle activity was increased in the ventral eye disc, as monitored by histone H3 phosphorylation (pH3) (*omb* ^*P7*^ */Y*, Fig. 2B, compare with wild type eye disc in 2A; quantified comparison in 2E) or BrdU incorporation (*omb* ^*P7*^ */Y*, Fig. 2D, compare with wild type eye disc in 2C) as markers for cell proliferation. There are two mitotic waves in the eye disc. The first mitotic wave occurs anterior to the MF and affects the cell population which will be recruited to form ommatidial clusters. Changing cell proliferation in the first mitotic wave will change the number of ommatidia [9,10]. *omb* hypomorphic eye discs showed an increase of mitosis in the first mitotic wave (Fig. 2B, D), as expected by their increase in ommatidial numbers. In contrast, the second mitotic wave occurs behind the MF and affects the number of cellular components assembled into the ommatidia [74]. Since *omb* is expressed in the anterior lateral margins, it is not expected to affect the second mitotic wave. This was confirmed in *omb* hypomorphic mutants (Fig. 2B, D) and by knocking down *omb* by GMR*>omb-RNAi*, which resulted in normally sized adult eyes (not shown). Therefore, *omb* appears to affect cell divisions in the undifferentiated region anterior to the MF.

We also analyzed the effect of *omb* loss of function mutant clones on cell proliferation. The *l(1)omb* ^*D4*^ null allele was used because it yielded stronger effects than the hypomorphic alleles In wild type eye discs, control clones (marked by lack of GFP) were of similar size relative to their twin spots, irrespective of location (Fig. 2F, G shows two different focal planes to allow clone size visualization and measurement in the infolded margins, in particular the "ventral flap"; data are summarized in Fig. 2J). As expected from the restricted *omb* expression pattern and from the phenotype of *omb* loss-of-function mutants, *omb* null mutant clones had a proliferative advantage relative to their twin spots (*omb* ^*+*^ */omb* ^*+*^) only in the ventral margin (Fig. 2I, arrow) but not in the center of the disc (Fig. 2H, arrow) or in the dorsal margin (Fig. 2I). *omb* clones in the ventral regions were about 3.5 times larger than *omb* clones in the central region of the disc or than wild type clones (summarized in Fig. 2J).

We next analyzed whether apoptosis plays a role in the *omb* over-expression phenotype. There is little cell death in wild type larval eye discs [75]. Before onset of retinal differentiation, *dpp-GAL4 c* ^*40*.*6*^ is expressed in the lateral and posterior margins; later it is restricted to the lateral eye disc margins. The expression in the lateral margins partially overlaps with the *omb* expression domain in the progenitor region (cf. S6 Fig.). Expression of *omb* driven by *dpp-GAL4* (*dpp>omb*) caused a strong reduction to total absence of the adult eye [52] and lack of retinal differentiation in the eye disc Enhanced apoptosis could be detected in the posterior margin of the *dpp>omb+GFP* eye disc (S3A. Fig.). Coexpressing the anti-apoptotic factor p35 (*dpp>omb+p35*) did not rescue adult eye size (data not shown) nor retinal differentiation in eye disc, although apoptosis was strongly reduced (S3B Fig.). These results suggest that apoptosis is not primarily responsible for eye size reduction at the larval and adult stages and that Omb mainly affects eye size by blocking cell proliferation.

### Omb can block retinal differentiation

**Fig 3:**
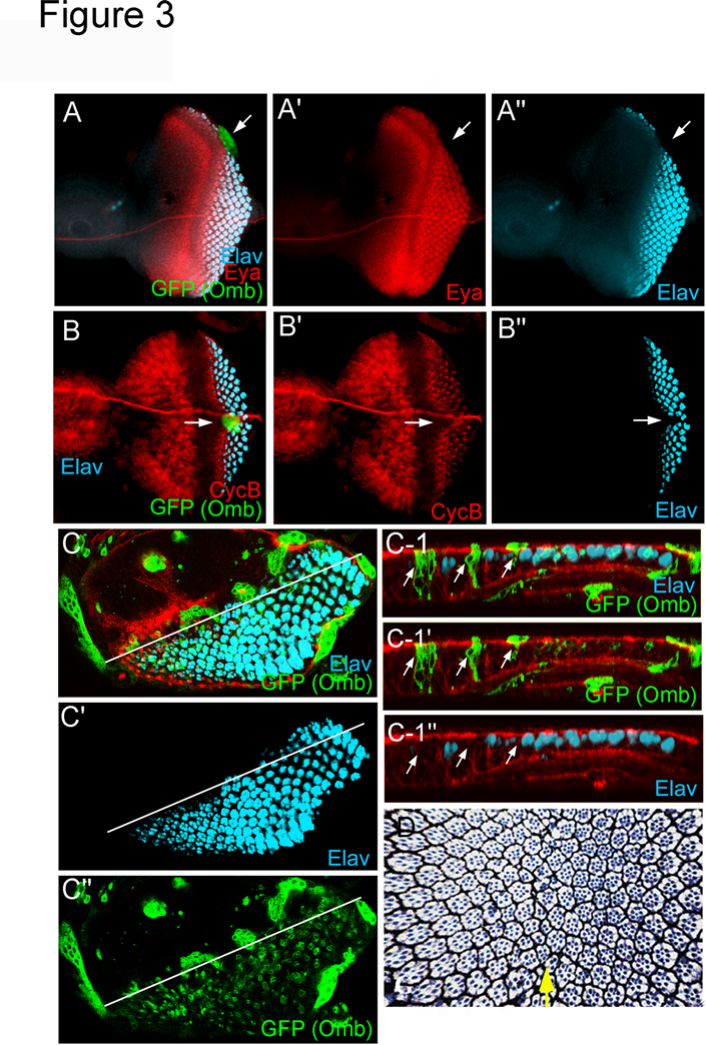
Ectopic *omb* expression can block morphogenetic furrow initiation, progression, and differentiation. Flip-out induced *omb* expression clones (*Act5C*>*omb*) marked by GFP coexpression repressed Elav (cyan) and Eya (red) expression. (A-A”) A clone at the posterior margin (arrow) inhibited MF initiation. (B-B”) A clone at the MF (arrow) inhibited MF progression (as indicated by CycB pattern, red) and neuronal differentiation (Elav, cyan). (C-C”) Omb expression level in *Act5C>omb* clones varied. Omb expression in a single ommatidial clusters (arrows) could autonomously block neuronal differentiation (Elav, cyan). The Z-section along the white line is shown in C-1 to C-1”. The relative level of Omb induction correlates to the signal of coexpressed GFP. (D) Tangential semi-thin sections through an adult eye of an *hs-omb* transgenic fly exposed to a single 1hr 37°C heat shock during mid-L3. Ommaditial patterning resumed normally beyond the dorso-ventral scar (arrow).

In addition to the effect on cell proliferation, Omb ectopic expression can block retinal differentiation. Ectopic clonal *omb* expression at the posterior margin prevented MF initiation (Fig. 3A). Ectopic clonal *omb* expression in the path of the MF blocked its progression (Fig. 3B) and neural differentiation (Fig. 3C-C-1”, arrow). Transient overexpression of *omb* by heat-induced expression of *hs-omb* in the entire eye field caused a dorso-ventral scar in the adult eye (Fig. 3D, arrow) characteristic of furrow-stop mutations [19]. Anterior and posterior to the scar, retinal differentiation proceeded normally, indicating that Omb does not irreversibly arrest MF progression and retinal differentiation. Previously we have shown that sustained *omb* expression posterior to the MF severely disturbs ommatidial development [52].

Increase in ommatidial number in *omb* hypomorphs apparently did not occur at the expense of gena tissue (the rim of naked tissue between retina and vibrissae) (Fig. 1B, C). This indicates that Omb in the lateral margins does not act to prevent spreading of eye fate into adjacent tissue domains. Rather, the increase in eye size in *omb* hypomorphs appears caused by overproliferation of retinal precursors in the ventral eye field (s. above).

### Omb inhibits cell proliferation by blocking Jak/STAT signaling through repression of *upd* transcription

To understand the mechanism by which Omb impedes proliferation in the ventral anterior eye disc, we tested the effect of Omb on the Upd/Jak/STAT signaling pathway, which promotes cell proliferation [7,9,10,18,24], as well as MF initiation [15,17,18,22,24].

**Fig 4:**
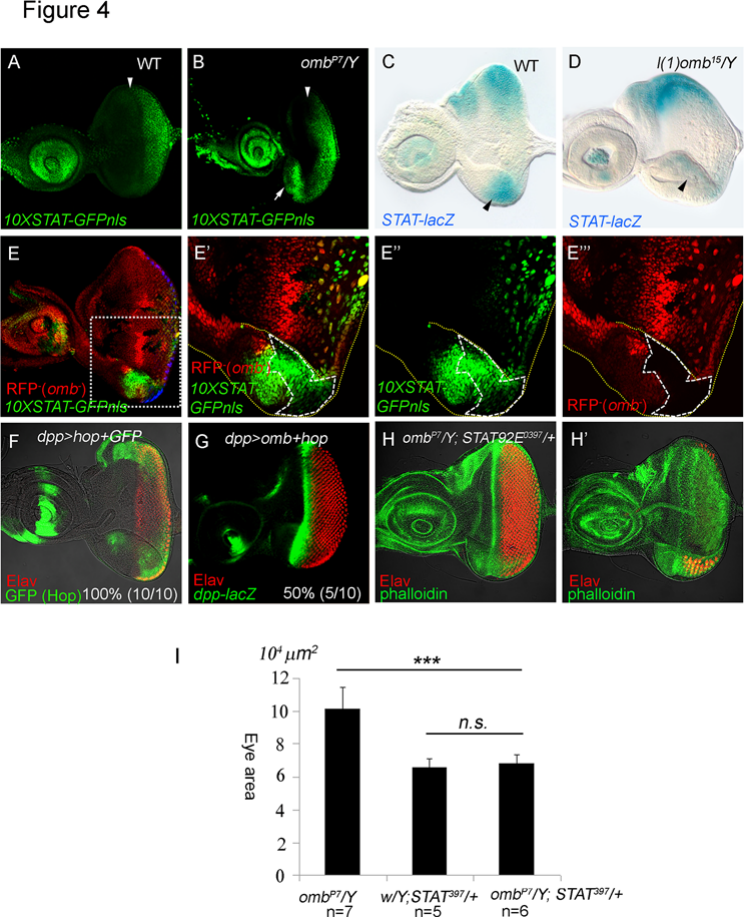
Omb blocks Jak/STAT signaling. 10XSTAT-GFP is a reporter of Jak/STAT signaling [76]. We added a nuclear localizing signal (nls) to obtain 10XSTAT-GFP-nls. (A) 10XSTAT-GFP-nls expression pattern (GFP, green) in wild type third instar eye disc. (B) The 10XSTAT-GFP-nls was ectopically expressed in the ventral eye margin (arrow) in an *omb* ^*P7*^ hypomorphic mutant eye disc. (A, B) The position of the MF, based on the DIC image, is marked by an arrowhead. (C) *STAT-lacZ* is repressed by Jak/STAT signaling. In wild type late third instar eye disc, its expression was strong in the lateral poles and weaker around the DV midline, as reported [57]. (D) In *l(1)omb* ^*15*^ */Y* eye discs, *STAT-lacZ* expression was attenuated in the ventral region. (E-E”’) *10XSTAT-GFP-nls* (green) was ectopically induced in *l(1)omb* ^*D4*^ mutant clones (clone marked by loss of RFP (red) expression and by dashed line). (E’-E”’) Higher magnification of the square marked in (E). *10XSTAT-GFP-nls* was non-autonomously induced by loss of *omb* in the ventral margin. (F) *dpp>hop+GFP* caused an enlargement of the eye disc (Elav, red; GFP, green). (G) Coexpression of *hop* with *omb* (*dpp>omb+hop*) could largely rescue the *dpp>omb* phenotype (*dpp-lacZ*, green; Elav, red). (H-H’) Reducing *STAT* dosage in *omb* ^*P7*^ */Y; STAT92E* ^*397*^ */+* larvae reduced the size of the ventral retinal field compared to that in *omb* ^*P7*^ */Y* (Fig. 2B). Different focal planes of *omb* ^*P7*^ */Y; STAT92E* ^*397*^ */+* were shown in H and H’. The quantified eye areas are summarized in (I).

The ligand Unpaired (Upd, FlyBase: *outstretched*, *os*) of the Jak/STAT pathway is expressed in the ventral eye disc at first instar and in the posterior center of the eye disc at second and early third instar [9,10,57]. Upd, acting through the Jak/STAT signaling pathway, promotes cell proliferation and represses *wg* transcription to promote MF initiation [7,9,10,18,24]. STAT signaling is induced by Upd and can be detected using STAT reporters containing STAT binding sites [9,10,18,24,76]. *Grh-STAT-lacZ* and *10X-STAT-GFP* reporter expression is high in the posterior region, consistent with STAT activity being induced by the Upd ligand [24]. *10X-STAT-GFP* is expressed in the posterior part of the second instar eye disc, before MF initiation. In the third instar eye disc, the 10XSTAT-GFP signal is much reduced and represents perdurance from earlier expression [76]. In the *omb* hypomorph *omb* ^*P7*^, STAT activity was ectopically activated in the ventral margin (Fig. 4B and S4 Fig.), as monitored by expression of 10XSTAT-GFP-nls (constructed in this study) which is normally expressed only in the posterior region of the eye disc (Fig. 4A). *omb* ^*P3*^ *>omb-RNAi* yielded similar results (not shown). A *STAT-lacZ* enhancer trap reporter, although not fully recapitulating the *STAT92E* mRNA pattern, is known to be negatively regulated by Jak/STAT activity [43,57] and, therefore, can be used as a reporter for Jak/STAT activity. In the wild type L3 eye disc, its expression was higher in the lateral poles and lower around the DV midline (Fig. 4C, see also [57]). In the *l(1)omb* ^*15*^ eye disc, *STAT-lacZ* expression was lost in the ventral region (Fig. 4D), suggesting an elevated STAT activity in this region. To determine whether *omb* regulates Jak/STAT activity cell-autonomously, we generated *l(1)omb* ^*D4*^ mutant clones. We found that 10XSTAT-GFP-nls was nonautonomously induced in ventral clones (Fig. 4E-E”’). These results suggest that Omb normally acts to repress Jak/STAT activity in the ventral region of the eye disc.

In order to determine, at which level of the Jak/STAT signaling cascade Omb inhibits this pathway, we coexpressed the Janus kinase gene *hopscotch* (*hop) with omb* (*dpp>omb+hop*) (Fig. 4G). Hop largely rescued retinal development indicating that Omb acts upstream of *hop*. *dpp>hop* (not shown) and *dpp>hop+GFP* (Fig. 4F) caused an enlargement of the eye disc, consistent with the role of Jak/STAT signaling in promoting cell proliferation. These results suggest that repression of Jak/STAT activity is a major mechanism by which ectopic Omb blocks eye development. We then asked whether increased Jak/STAT signaling can account for the *omb* loss-of-function phenotype. We reduced the *STAT92E* dose in the *omb* ^*P7*^ mutant background, (*omb* ^*P7*^ */Y; STAT* ^*06346*^ */+* and *omb* ^*P7*^; *STAT92E* ^*397*^ */+*), which caused attenuation of the ventral outgrowth of eye disc (Fig. 4H, H’, two focal planes; quantified comparison in 4I). These results suggest that the overgrowth phenotype elicited by reduced *omb* expression requires Jak/STAT activity. Therefore, in its normal function Omb appears to suppress inappropriate Jak/STAT signaling at the ventral margin.

**Fig 5:**
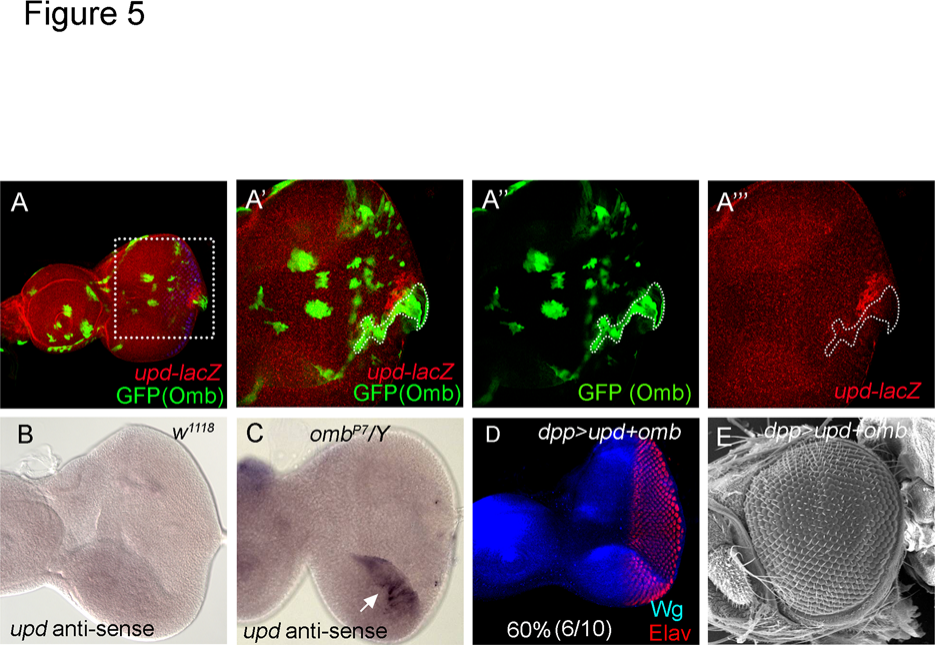
*omb* can repress *upd* transcription. (A-A”’) *omb* expression clone induced by *Act5C-GAL4* suppressed *upd-lacZ* expression (*Act5C>omb+GFP* in *upd-lacZ*). (A’-A”’) are a higher magnification of the area marked in (A). GFP (green) marks the Omb expressing cells. Omb expressing cells suppressed *upd-lacZ* (red). (B-C) RNA *in situ* hybridization of third instar eye discs. (B) In late third instar, no signal was detected by *upd* anti-sense probe in wild type eye disc. (C) *upd* mRNA was ectopically expressed in the ventral eye margin of *omb* ^*P7*^ */Y*. (D, E) Coexpression of *omb* with *upd* (in *dpp>omb+upd*) partially or fully rescued retinal development in the eye disc (D) and adult eye (E). (Elav, red; Wg, blue)

Upd is the ligand of Jak/STAT pathway and expressed in the center of the posterior margin of the eye disc in L2 and L3 larvae (S5A-D, F Fig.; [9,57]). We examined whether *omb* could suppress Jak/STAT activity by downregulating *upd* expression. Expression of *omb* along the posterior margin (in *dpp>omb+GFP*) suppressed *upd-lacZ* expression (S5E, E’ Fig.). Clonal expression of *omb* repressed *upd-lacZ* cell autonomously (Fig. 5A-A”’). We further tested whether *upd* expression is affected in *omb* mutant eye discs. We performed RNA *in situ* hybridization on wild type and *omb* ^*P7*^ eye discs and found that *upd* was derepressed in the ventral margin in *omb* ^*P7*^ (Fig. 5C) compared wild type (Fig. 5B). Thus, ectopically expressed Omb can suppress *upd* transcription, and Omb in its normal expression domain restricts *upd* transcription in the ventral eye margin. Moreover, coexpression of *omb* with *upd* (in *dpp>omb+upd*) largely rescued retinal development in the eye disc (Fig. 5D) and the adult eye (Fig. 5E). These results suggest that Omb, in its endogenous expression domain, acts by repressing *upd*, thus limiting cell proliferation in the ventral eye. Our results further suggest that repression of Jak/STAT signaling occurs at the transcriptional level of *upd* and is the major mechanism by which Omb blocks cell proliferation in eye development.

### Omb is a mediator of Wg anti-retinal function

**Fig 6:**
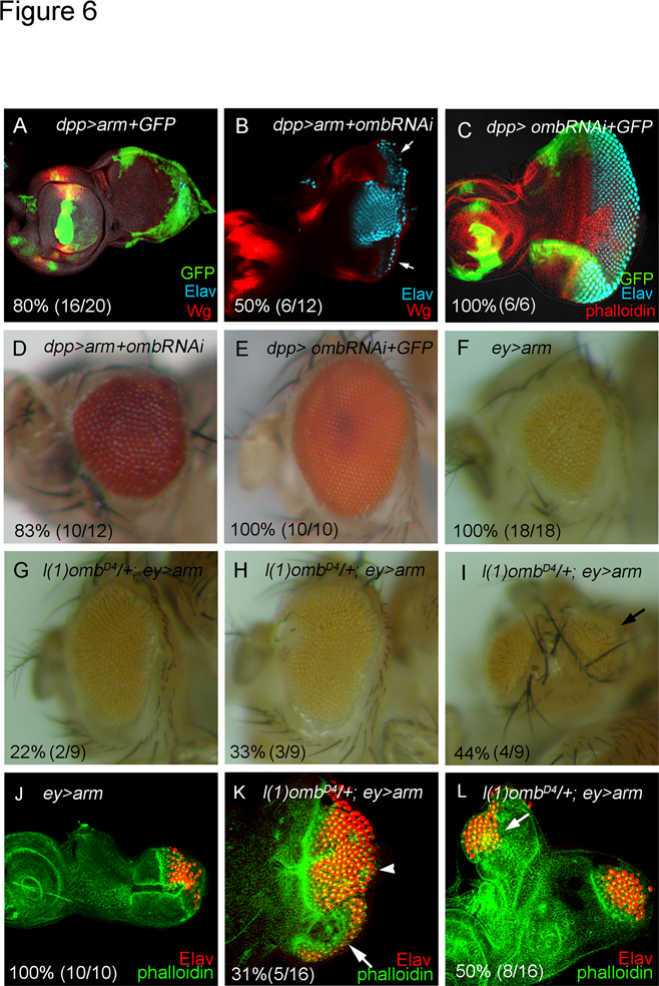
Functional relationship between *wg* and *omb*. (A) *dpp>arm*+GFP eye discs were reduced in size and showed no neuronal differentiation (Wg, red; Elav, cyan; GFP, Green). (B, D) *dpp>arm+omb-RNAi* caused partial rescue of eye disc size and neuronal differentiation (B) and adult eye (D). (C, E) *dpp>omb-RNAi+GFP* did not affect retinal development in eye disc (C) and adult eye (E). Misexpression of *arm* by *ey*-*GAL4* (*ey>arm*) caused eye size reduction in adult eye (F) and in eye disc (J). Reduction of *omb* genetic dosage (*l(1)omb* ^*D4*^ */+*) in the background of *ey*>*arm* partially rescued the eye size with full penetrance in adult (G-I) and in eye disc (K, L). 31% of these eye discs showed ventral expansion of retinal differentiation. Interestingly, 44~50% of *l(1)ombD4/+; ey>arm* flies have dorsal ectopic eyes in adult (I) and in eye discs (6L).

Since Wg and Omb are expressed in a similar pattern and both block retinal development, we asked whether the anti-retinal activity of the Wg signal is mediated by Omb. The Wg effector Arm was expressed to mimic Wg signaling. In *dpp>arm+GFP*, eye disc size was reduced and no neuronal differentiation was detected (Fig. 6A), consistent with a block of eye development by Wg signaling. *dpp* expression along the posterior and lateral margins has little overlap with endogenous *wg* expression (S6A’-E’, A”-E” Fig.). Therefore, this experiment tests the effect of ectopic Wg signaling. When *omb* was knocked down in the background of *dpp>arm* (in *dpp>arm+omb-RNAi*), disc size and neuronal differentiation were partly recovered (Fig. 6B). Adult eye size also was largely restored (Fig. 6D). Knock down of Omb in the *dpp* expression domain (*dpp>omb-RNAi+GFP*) caused no significant effect on retinal development (Fig. 6C, E). Misexpression of *arm* by *ey*-*GAL4* (*ey>arm*) caused a reduction of adult eye size and retinal differentiation in eye disc (Fig. 6F, J). When *omb* dosage was reduced (*l(1)omb* ^*D4*^/+), the *ey>arm* phenotype was partially rescued with full penetrance (Fig. 6G-I, K-L). These results suggest that Omb is one of the mediators of Wg activity in blocking eye development.

## Discussion

### *omb* represses retinal development

In this study, we demonstrate that *omb* is a negative regulator of retinal development. Omb can block eye development at several levels: cell proliferation, MF initiation, and progression. In *omb* loss-of-function mutant or RNAi-knockdown animals, the most prominent phenotype was an enlargement of the ventral eye (Fig. 1) due to extra cell proliferation (Fig. 2). *omb* mutant clones in its expression domain at the ventral margin were 3.5 times larger than control clones (Fig. 2). These loss-of-function results were supported by the opposite effect in gain-of-function experiments. In *omb* gain-of-function animals, the size of eye disc (S1C Fig.) and adult eye was reduced (data not shown). In addition, *omb*-expressing clones blocked MF initiation (Fig. 3A) and progression (Fig. 3B). Expression of *omb* along the margins (*dpp>omb+GFP*) could completely block retinal development (S3B-C Fig.).

*Act5C>omb* clones were not detected in late third instar eye discs. Rare clones were observed only when larvae were raised at 17°C and examined at early to mid-third instar (Fig. 4). These clones were often round and sorted out from the neuroepithelial layer. This behavior has previously been observed for *omb* gain-of-function clones in the wing imaginal disc [49].

We identified the Jak/STAT signaling pathway asdownregulated by Omb. In *omb* loss-of-function animals, the *10XSTAT-GFP-nls* and *upd-lacZ* expression were elevated in the ventral region (Figs. 4B, 5C). Conversely, when *omb* was ectopically expressed in the posterior and lateral margins, *upd-lacZ* expression at the margins was reduced (S5E, E’ Fig.). These results show that *upd* transcription is repressed by Omb in the lateral margins, especially the ventral margin.

The repression of the Upd signaling cascades by Omb is of developmental relevance. Ectopic expression of *omb* in the margins (*dpp>omb+GFP*) blocked retinal development (S3C,5E Figs.). Coexpression with of *hop* (*dpp>omb+hop*) could nearly fully restore retinal development in the eye disc (Fig. 4G). Reducing the dosage of STAT (in *omb* ^*P7*^ */Y; STAT92E* ^*397*^ */+*) partially suppressed the *omb* ^*P7*^ enlarged eye phenotype (Fig. 4H), suggesting that STAT signaling is downstream of *omb* and involved in causing its mutant phenotype. These results indicate that the repression of the Upd/Jak/STAT pathways is responsible for the block of retinal development by Omb.

*upd* is transcriptionally repressed by Omb. Is it a direct transcriptional target of Omb? Omb is a transcription factor of the T-box family all members of which bind to a common consensus element, the T-box binding element (TBE, [77]). Based on transcript microarray data and cell culture studies, Omb acts predominantly as a transcriptional repressor ([52]; A. Klebes and G. O. Pflugfelder, unpublished data). A bioinformatic search using a position weight matrix constructed from bona fide TBEs identified a well conserved high-affinity potential TBE in the upstream region of *upd* (G. O. Pflugfelder, unpublished data). This finding and the cell-autonomous repression of *upd* by Omb (Fig. 5A) as well as the derepression of *upd* in the ventral *omb* domain in *omb* mutant eye discs (Fig. 5C) suggest that repression of *upd* by Omb may be direct. However, Omb cannot be the only factor that prevents *upd* expression from the lateral margins, because dorsally *upd* is not derepressed in *omb* mutant eye discs.

### Omb partially mediates the anti-retinal function of Wg signaling

Since Wg and Omb are expressed in similar patterns and since both can block retinal development at multiple steps, the question arises whether Omb mediates Wg signaling. We found that the anti-retinal effects of ectopic Wg signaling (*ey>arm* and *dpp>arm*) were attenuated when the *omb* dosage was reduced. The partial rescue suggests that Omb mediates part of the Wg effect and that additional factor(s) are likely to be involved. Our analyses show that the effect of Omb is partly similar to that of Wg and partly different (Fig. 6).

A clear difference was the opposite effect on cell proliferation. Omb repressed cell proliferation, through a block of Upd/Jak/STAT signaling. In contrast, enhanced Wg signaling (in *axin* mutant) causes overgrowth [78], whereas loss of Wg results in reduction of eye disc size [30,35]. Therefore, Wg can promote cell proliferation.

Loss of *omb* affected primarily development of the ventral eye margin. The ventral bias included effects on STAT activity. Therefore, the effect of *wg* in the dorsal side may be mediated by another factor, either independent of Omb or functionally redundant with Omb. Dorsal eye fate is governed by the expression of members of the *Iroquois* gene complex (*Iro-C*)[79]. Its activity modulates the function of genes that are symmetrically expressed at the poles of the eye disc, like *teashirt*, for instance [79,80]. The specification of dorsal rim ommatidia late in eye development provides an example of how Omb function is modulated at the lateral eye margins.

During pupal eye development, a dorsal Wg gradient specifies three cell fates at the dorsal eye boundary: pigment rim, polarization-sensitive dorsal rim (DR) ommatidia, and bald ommatidia (lacking the mechanosensory hairs). Omb which is induced by Wg, is sufficient to induce the DR fate in the dorsal eye. Ectopic *omb* induces the dorsally restricted expression of *homothorax* (*hth*) which together with ubiquitous *extradenticle* (*exd*) allows the formation of Hth/Exd complexes which specify DR development [81]. Dorsalisation of the ventral eye by ubiquitous expression of *Iro-C* genes causes DR fate also along the ventral margin. The monopolar *Iro-C* expression thus determines the different functional outcome of symmetrical *omb* expression at the two margins. Like in early eye development, loss of *omb* has little consequence for dorsal eye development during specification of DR ommatidia. The discrepancy between strong effect in *omb* gain-of-function and little effect in *omb* loss-of-function dorsal eye phenotypes has been attributed to a redundant function exerted by the related T-box genes *Dorsocross* (*Doc*) [82].

Intriguingly, the closely *omb*-related vertebrate T-box genes Tbx2/3/5 are all expressed in a polar pattern, first in the dorsal eye cup and later in the dorsal retina [83,84,85,86]. A role of human TBX5 in eye development is apparent from the frequent ophtalmological symptoms of patients suffering from Holt Oram Syndrome (TBX5 haploinsufficiency) [87,88]. The related expression patterns of Tbx2/3/5 and *omb* in eye development suggest conservation of ancient functions which were already present in an evolutionary precursor before the split into the protostome and deuterostome lineages [89], in spite of the widely differing mechanisms of eye ontogenesis in metazoans [90,91].

## Supporting Information

S1 Fig Reduced Omb expression by *omb* ^*P3*^ *>ombRNAi* and increased Omb expression in the *omb* ^*For*^ mutant. Eye-antennal discs stained with anti-Omb (green) and anti-Elav (red). Marginal expression is indicated by arrows, expression in retinal basal glial cells by arrowheads. (A) wild type, (B) *omb* ^*P3*^ *>omb-RNAi*, (C) *omb* ^*For*^, (D) *omb* ^*P3*^ *>GFP* and (E) *omb* ^*P7*^ *>GFP*. In *omb* ^*For*^, Omb is increased in the dorsal and ventral margin and in the retinal basal glia. The disc size is reduced (C). The marginal expression of *omb* ^*P7*^-*GAL4* was broader than that of *omb* ^*P3*^-*GAL4*. In (C) and (D) the retinal basal glial cells were below the focal plane.
(TIF)

S2 Fig Loss of *omb* causes primarily ventral overgrowth. (A-D) The boundary of dorsal/ventral fields in third instar eye discs was monitored by the position of the optic stalk (white arrowhead), anti-Bar antibody staining (A, B) and the ventrally expressed *fng-lacZ* (C, D). Dotted lines mark the MF and the projection from the optic stalk entry point onto the MF. In (A, B) the dotted line also visualises the line of mirror symmetry in the Bar expression pattern. The BarH1 and BarH2 expression in photoreceptor cells R1 and R6 [71] is mirror-symmetrical with regard to the equator. The solid line in (C, D) marks the dorsal boundary of the ventral *fng-lacZ* expression domain. (A) *w* ^*1118*^ */Y*, (B) *omb* ^*P7*^ */Y*, (C) *fng-lacZ* and (D) *omb* ^*P7*^ */Y; fng-lacZ*. The D/V eye field was symmetrical in the third instar eye disc of wild type, but the ventral field was expanded in *omb* ^*P7*^ */Y*. (E) The number of rows of ommatidia in each eye disc, and the numbers of ommatidia in the dorsal and ventral eye fields were counted at different stages of eye disc development. In wild type eye disc, the dorsal and ventral eye fields were always of equal size. In *omb* ^*P7*^ */Y*, the ventral eye field was consistently larger than the dorsal field.
(TIF)

S3 Fig Coexpression of the cell death inhibitor p35 does not rescue eye development blocked by Omb. (A) *dpp-GAL4* driven GFP (*dpp>GFP*) expression (GFP, green) overlapped with the *omb-lacZ* expression (red) domain in the lateral margins. In early eye disc, *dpp-GAL4* expression is similar to that of *dpp-lacZ* (S6 Fig.) in the posterior and lateral margins. Unlike *dpp-lacZ*, *dpp-GAL4* is not expressed in the progressing MF in mid to late third instar eye disc. (B) *dpp>omb+GFP*, as *dpp>omb*, completely blocked eye development in adult (B) and in late third instar eye disc (C). The eye disc has no neuronal differentiation (Elav, blue) but has elevated activated caspase 3 (red). (D) Blocking apoptosis by coexpression of *p35 (dpp>omb+p35)* significantly reduced the caspase 3 signal but did not rescue eye size or retinal differentiation. Scale bar: 50um.
(TIF)

S4 Fig The Jak/STAT activity is detected in the ventral margin of *omb* mid- and late third instar eye discs. 10XSTAT-GFPnls is a Jak/STAT reporter. (A-C) Jak/STAT activity in *omb* ^*P7*^ eye discs. (A-A”) 10XSTAT-GFPnls was found in the posterior eye field in the early third instar eye disc of *omb* ^*P7*.^ (B-B”) Jak/STAT activity was activated in posterior eye field and ventral margin (arrow) of mid-third instar larvae. (C, C”) Jak/STAT activity was detected in the posterior eye field as well as ventral margin (arrow) in the late third eye field. Elav (red), GFP (green).
(TIF)

S5 Fig Expression of *omb* suppresses *upd* transcription. (A-C’) The expression pattern of *omb* ^*P1*^-*lacZ* (red) and *upd>GFP* (green) did not overlap in late second (A), early third (B) and late-third instar eye discs (C). *omb* ^*P1*^-*lacZ* is also expressed in the retinal basal glia which lies at the basal surface and does not overlap with the *upd* expressing cells in the neuroepithelial layer (not shown). (D) The expression pattern of *upd-lacZ (red)* in wild type. Elav (cyan). (E, E’) *dpp>omb+GFP* (GFP, green) suppressed *upd-lacZ* expression (red) at the center of the posterior margin (arrow)
(TIF)

S6 Fig Relative expression pattern of *omb*, *dpp* and *wg* during eye disc development. (A-E) The expression patterns of *omb* (visualized by *omb* ^*P3*^ *>GFP*, green), (A’-E’) Wg (anti-Wg, red), and (A”-E”) *dpp* (represented by *dpp-lacZ*, blue) were followed during eye-antennal disc development from early second instar to late third instar. (A”’-E”’) shows the merge images of *omb* ^*P3*^ *>GFP*, *dpp-lacZ* and *Wg* immunostaining.
(TIF)
