# Supplementary figures and images for: Optomotor-Blind Negatively Regulates Drosophila Eye Development by Blocking Jak/STAT Signaling
Source: PLoS One. 2015 Mar 17;10(3):e0120236. doi: 10.1371/journal.pone.0120236 (PMC4363906; doi:10.1371/journal.pone.0120236)

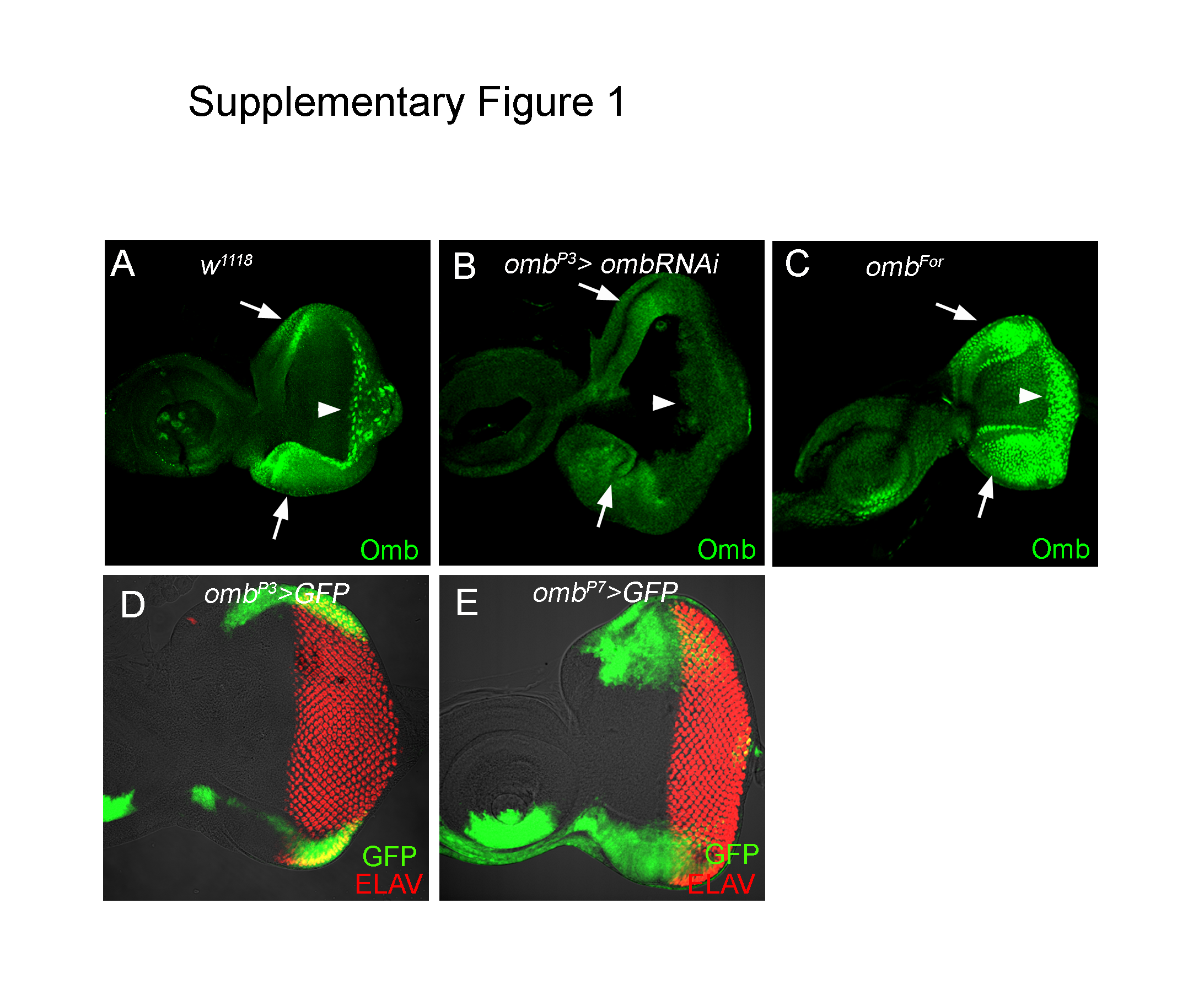

Supplement: S1 Fig — Eye-antennal discs stained with anti-Omb (green) and anti-Elav (red). Marginal expression is indicated by arrows, expression in retinal basal glial cells by arrowheads. (A) wild type, (B) omb P3 >omb-RNAi, (C) omb For, (D) omb P3 >GFP and (E) omb P7 >GFP. In omb For, Omb is increased in the dorsal and ventral margin and in the retinal basal glia. The disc size is reduced (C). The marginal expression of omb P7-GAL4 was broader than that of omb P3-GAL4. In (C) and (D) the retinal basal glial cells were below the focal plane. (TIF) [file pone.0120236.s001.tif]

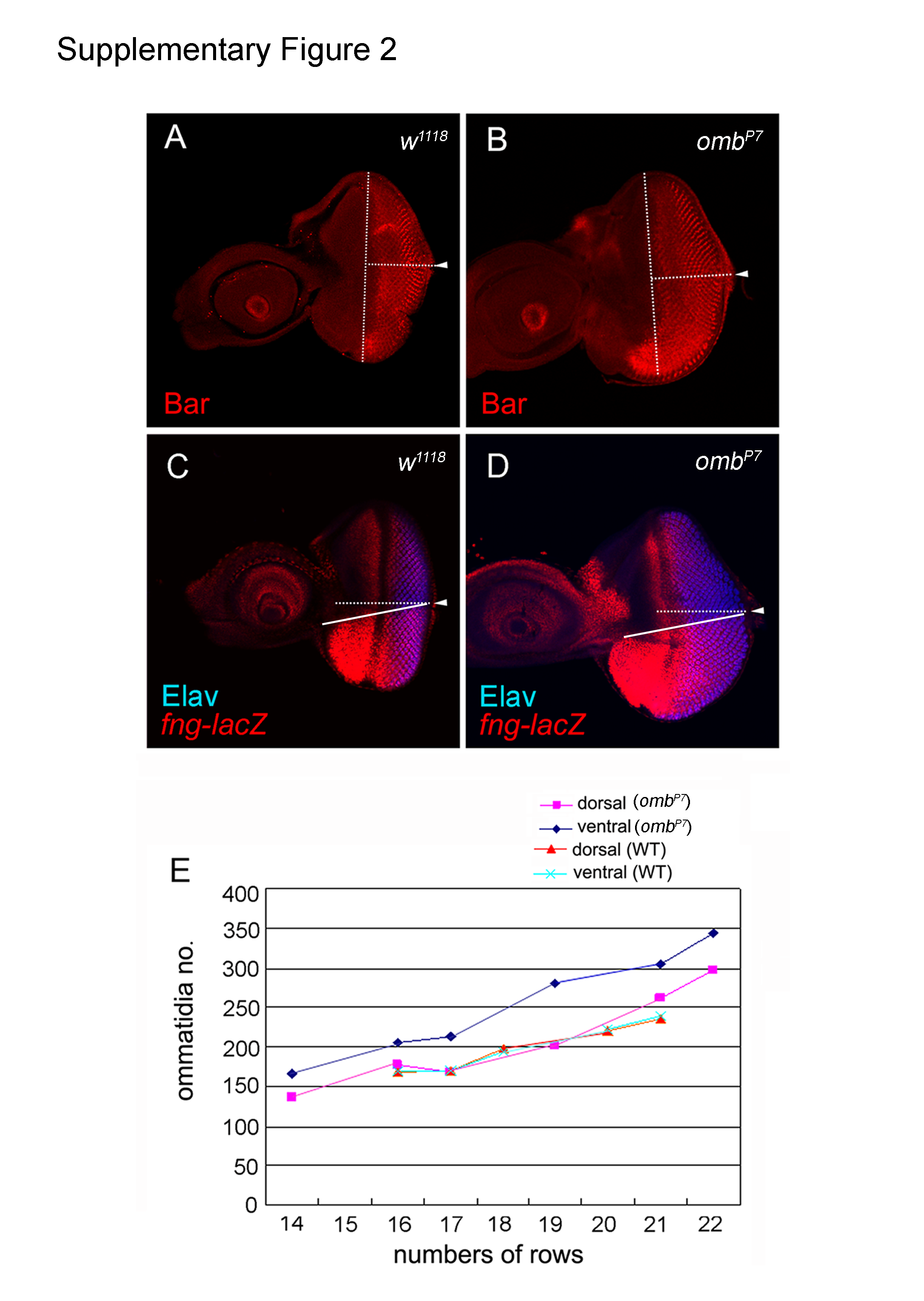

Supplement: S2 Fig — (A-D) The boundary of dorsal/ventral fields in third instar eye discs was monitored by the position of the optic stalk (white arrowhead), anti-Bar antibody staining (A, B) and the ventrally expressed fng-lacZ (C, D). Dotted lines mark the MF and the projection from the optic stalk entry point onto the MF. In (A, B) the dotted line also visualises the line of mirror symmetry in the Bar expression pattern. The BarH1 and BarH2 expression in photoreceptor cells R1 and R6 [71] is mirror-symmetrical with regard to the equator. The solid line in (C, D) marks the dorsal boundary of the ventral fng-lacZ expression domain. (A) w 1118 /Y, (B) omb P7 /Y, (C) fng-lacZ and (D) omb P7 /Y; fng-lacZ. The D/V eye field was symmetrical in the third instar eye disc of wild type, but the ventral field was expanded in omb P7 /Y. (E) The number of rows of ommatidia in each eye disc, and the numbers of ommatidia in the dorsal and ventral eye fields were counted at different stages of eye disc development. In wild type eye disc, the dorsal and ventral eye fields were always of equal size. In omb P7 /Y, the ventral eye field was consistently larger than the dorsal field. (TIF) [file pone.0120236.s002.tif]

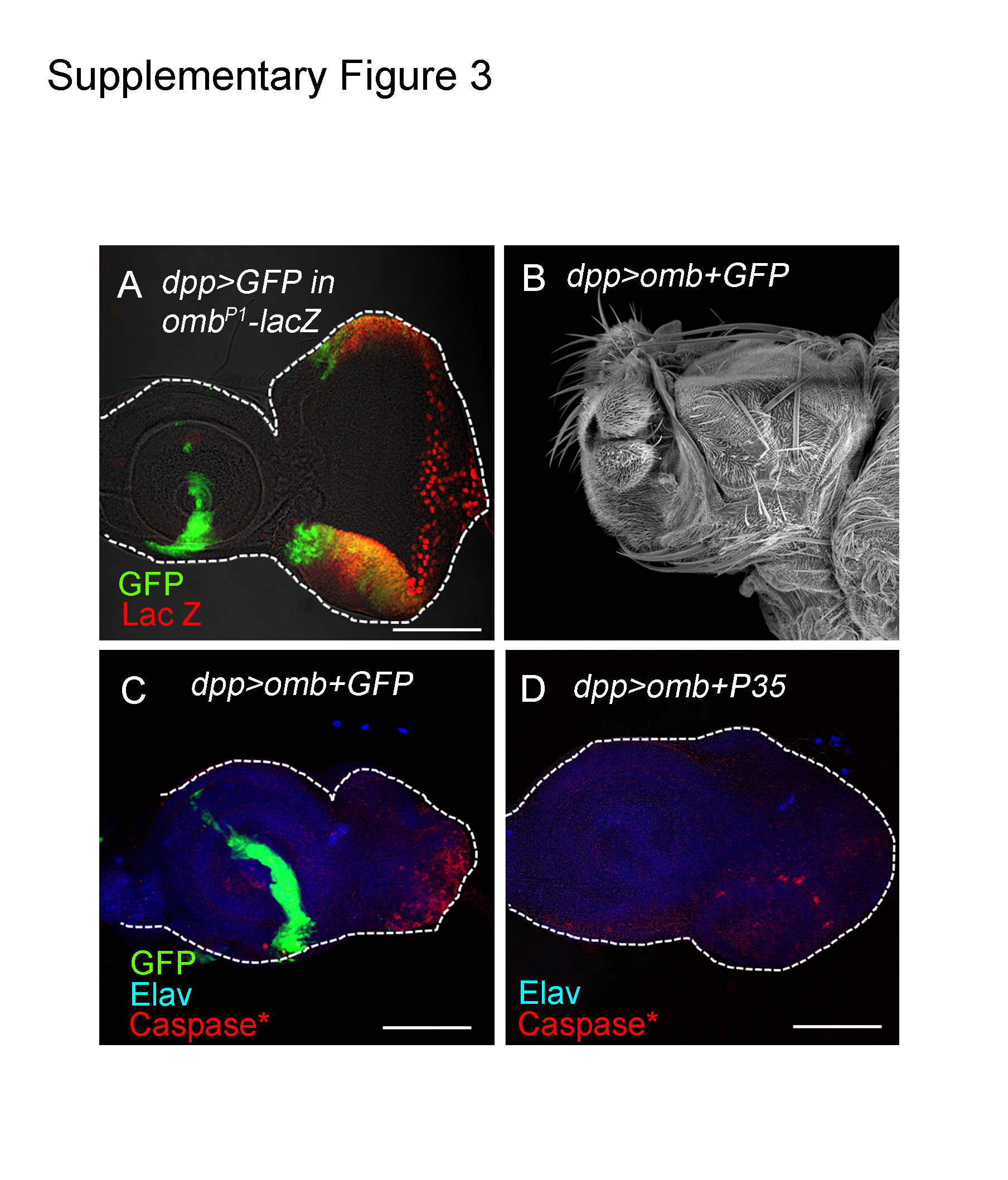

Supplement: S3 Fig — (A) dpp-GAL4 driven GFP (dpp>GFP) expression (GFP, green) overlapped with the omb-lacZ expression (red) domain in the lateral margins. In early eye disc, dpp-GAL4 expression is similar to that of dpp-lacZ (S6 Fig.) in the posterior and lateral margins. Unlike dpp-lacZ, dpp-GAL4 is not expressed in the progressing MF in mid to late third instar eye disc. (B) dpp>omb+GFP, as dpp>omb, completely blocked eye development in adult (B) and in late third instar eye disc (C). The eye disc has no neuronal differentiation (Elav, blue) but has elevated activated caspase 3 (red). (D) Blocking apoptosis by coexpression of p35 (dpp>omb+p35) significantly reduced the caspase 3 signal but did not rescue eye size or retinal differentiation. Scale bar: 50um. (TIF) [file pone.0120236.s003.tif]

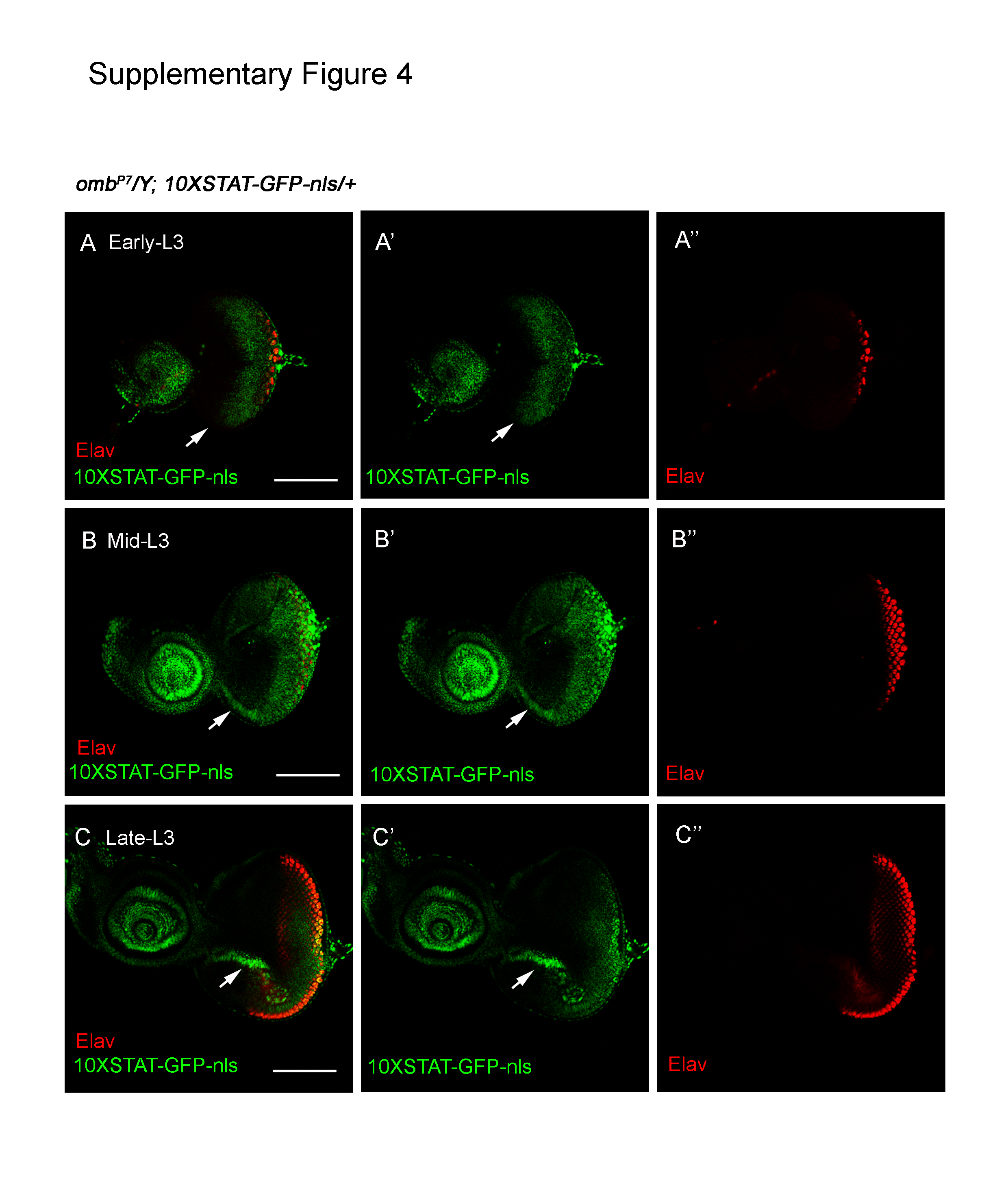

Supplement: S4 Fig — 10XSTAT-GFPnls is a Jak/STAT reporter. (A-C) Jak/STAT activity in omb P7 eye discs. (A-A”) 10XSTAT-GFPnls was found in the posterior eye field in the early third instar eye disc of omb P7. (B-B”) Jak/STAT activity was activated in posterior eye field and ventral margin (arrow) of mid-third instar larvae. (C, C”) Jak/STAT activity was detected in the posterior eye field as well as ventral margin (arrow) in the late third eye field. Elav (red), GFP (green). (TIF) [file pone.0120236.s004.tif]

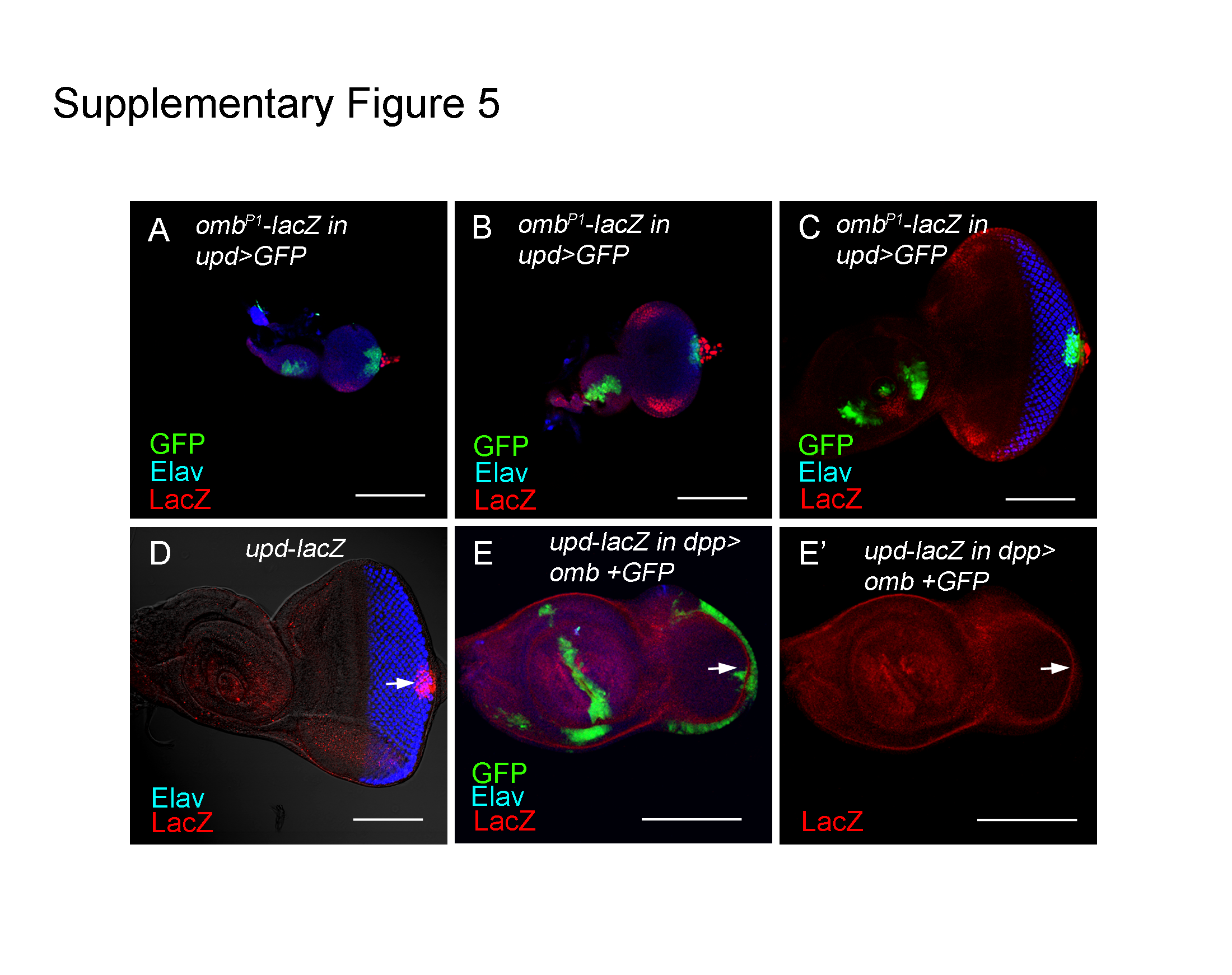

Supplement: S5 Fig — (A-C’) The expression pattern of omb P1-lacZ (red) and upd>GFP (green) did not overlap in late second (A), early third (B) and late-third instar eye discs (C). omb P1-lacZ is also expressed in the retinal basal glia which lies at the basal surface and does not overlap with the upd expressing cells in the neuroepithelial layer (not shown). (D) The expression pattern of upd-lacZ (red) in wild type. Elav (cyan). (E, E’) dpp>omb+GFP (GFP, green) suppressed upd-lacZ expression (red) at the center of the posterior margin (arrow) (TIF) [file pone.0120236.s005.tif]

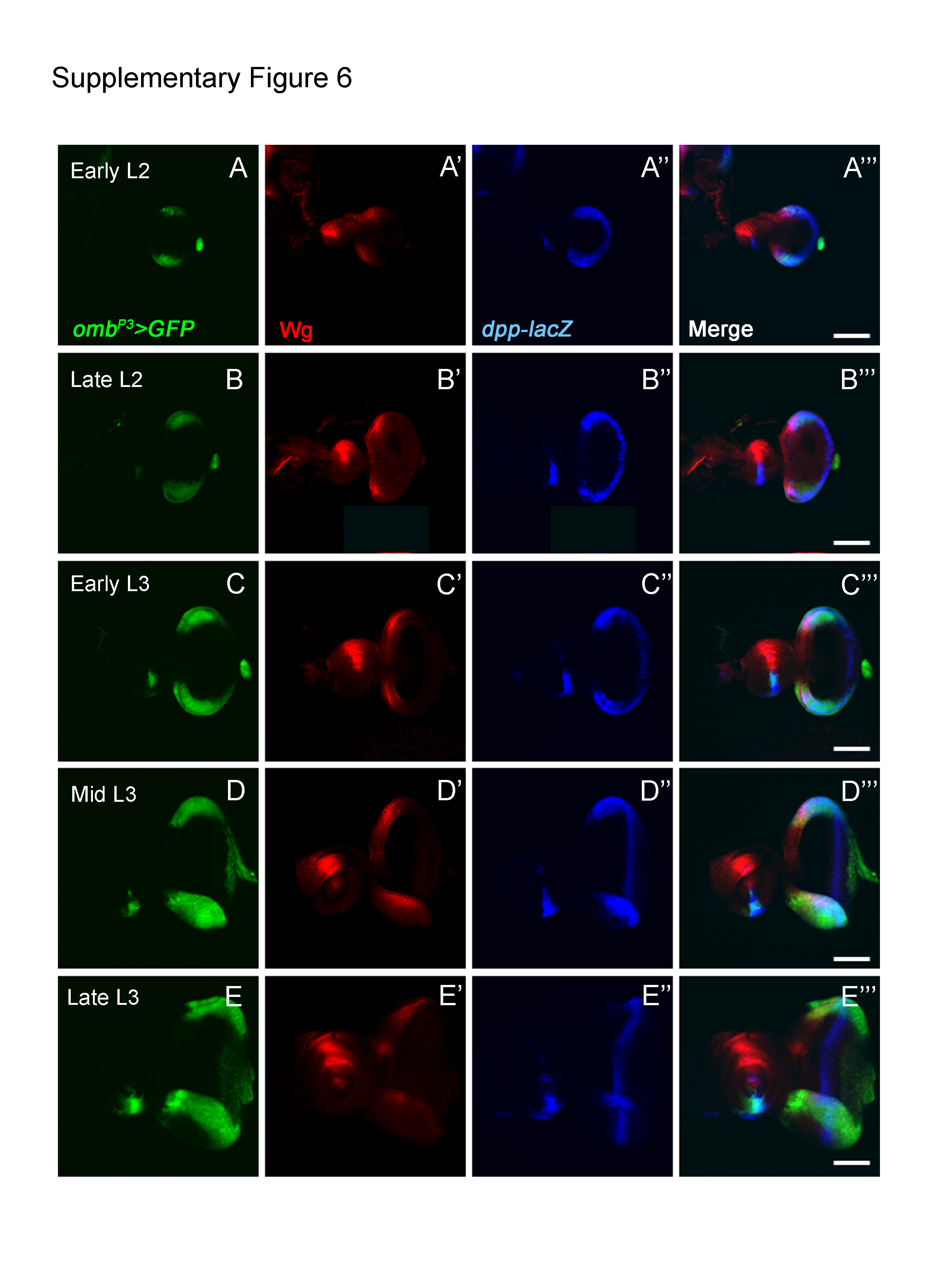

Supplement: S6 Fig — (A-E) The expression patterns of omb (visualized by omb P3 >GFP, green), (A’-E’) Wg (anti-Wg, red), and (A”-E”) dpp (represented by dpp-lacZ, blue) were followed during eye-antennal disc development from early second instar to late third instar. (A”’-E”’) shows the merge images of omb P3 >GFP, dpp-lacZ and Wg immunostaining. (TIF) [file pone.0120236.s006.tif]
